# Does sit-to-stand transition velocity vary across the day? Association with physical functioning and fatigability in community-dwelling older adults

**DOI:** 10.1093/geroni/igag040

**Published:** 2026-04-16

**Authors:** Antti Löppönen, Laura Karavirta, Merja Rantakokko, Katja Lindeman, Christophe Delecluse, Evelien Van Roie, Taina Rantanen, Lotta Palmberg

**Affiliations:** Faculty of Sport and Health Sciences and Gerontology Research Center, University of Jyväskylä, Jyväskylä, Finland; Faculty of Sport and Health Sciences and Gerontology Research Center, University of Jyväskylä, Jyväskylä, Finland; Faculty of Sport and Health Sciences and Gerontology Research Center, University of Jyväskylä, Jyväskylä, Finland; The Wellbeing services county of Central Finland, Jyväskylä, Finland; Faculty of Sport and Health Sciences and Gerontology Research Center, University of Jyväskylä, Jyväskylä, Finland; Department of Movement Sciences, Physical Activity, Sports and Health Research Group, KU Leuven, Leuven, Belgium; Department of Movement Sciences, Physical Activity, Sports and Health Research Group, KU Leuven, Leuven, Belgium; Faculty of Rehabilitation Sciences, REVAL-Rehabilitation Research Center, University of Hasselt, Diepenbeek, Belgium; Faculty of Sport and Health Sciences and Gerontology Research Center, University of Jyväskylä, Jyväskylä, Finland; Faculty of Sport and Health Sciences and Gerontology Research Center, University of Jyväskylä, Jyväskylä, Finland; Department of Public Health, University of Turku and Turku University Hospital, Turku, Finland

**Keywords:** Accelerometer, Biomarker, Chair rise, Wearable, Physical activity

## Abstract

**Background and Objectives:**

Physical fatigability increases with age and may act as a barrier to activity and a marker of functional decline. In older adults, strength-demanding, device-based variables such as free-living sit-to-stand (STS) transitions may be associated with physical fatigability. This study examines STS velocity patterns across a 24-hr cycle and their associations with physical fatigability and functioning in older adults.

**Research Design and Methods:**

This cross-sectional study included a population-based sample of 75-, 80-, and 85-year-old people (*n *= 479, 60% women). STS transitions were recorded for 3–7 days using a thigh-worn accelerometer, with velocities summarized in two-hour intervals and normalized to daily means. Participants were grouped by self-reported walking fatigability, performance fatigability, and physical functioning. Group differences were analyzed using non-parametric tests and logistic regression, adjusted for age cohort and sex.

**Results:**

Individuals with high self-reported walking fatigability showed greater declines in free-living STS angular velocities in the late afternoon (4–6 p.m.: odds ratio [OR] = 1.39 per 5% decrease, *p *= .009) than those with low fatigability. Similarly, individuals with high performance fatigability exhibited greater velocity declines in the afternoon and early evening (2–4 p.m.: OR = 1.19, *p *= .034; 6–8 p.m.: OR = 1.15, *p *= .035). Evening declines were also most pronounced among those with physical functioning limitations (6–8 p.m.: OR = 1.19, *p *= .028; 8–10 p.m.: OR = 1.21, *p *= .003).

**Discussion and Implications:**

Measuring STS velocity decline may help in the identification of older individuals with higher fatigability and poorer physical functioning and may open new possibilities for wearable-based remote monitoring and individualized care.

Innovation and Translational Significance: Wearable technologies may offer a new way to investigate physical fatigability in free-living environments by capturing repeated daily activities such as STS transitions. This approach is innovative in that it shifts fatigability assessment from controlled laboratory settings to natural, everyday contexts, potentially enabling more personalized and timely monitoring. Such continuous observation could also support the development of remote rehabilitation and home-based care. However, it remains to be clarified what role fatigability plays in the observed declining trends in STS transition velocity throughout the day.

Fatigue is a significant concern for older adults that plays a central role in functional decline. From a physiological perspective, fatigue reflects age-related alterations across multiple bodily systems that reduce physiological reserve and increase the effort required to perform everyday movements, thereby contributing to declines in mobility and physical function (Avlund, 2010; Schrack et al., 2010). Consequently, fatigue not only acts as a barrier to physical activity but may also serve as a mechanism in which performing routine daily tasks and maintaining independence become increasingly challenging (Avlund, 2010; Barbosa et al., 2016; Egerton et al., 2016; Kowal & Fortier, 2007). Research on fatigue, however, is limited because individuals behaviorally adapt and self-pace to maintain fatigue at an acceptable level, which can lead to similar reported fatigue despite substantial differences in activity or workload. Fatigability, defined as fatigue experienced in relation to standardized tasks, addresses this limitation by situating fatigue within the context of activity demands (Eldadah, 2010). As such, fatigability has emerged as a promising indicator of phenotypic aging (Schrack et al., 2020a), and has been linked to functional decline, reduced physical activity, and mortality in older adults (Glynn et al., 2022; Simonsick et al., 2016; Wanigatunga et al. 2018). Consistent with this framework, recent evidence suggests that fatigability may mediate the relationship between poorer physical capacity and low physical activity in later life, supporting its role as an important contributor to age-related declines in physical function and activity levels (Koivunen et al., 2025).

In free-living environments, fatigability has been studied in relation to wearable sensors by examining activity counts/volume, activity fragmentation, and 24-hr rest–activity rhythm patterns (Graves et al., 2021; Moored et al., 2022; Palmberg et al., 2020; Schrack et al., 2019). Research has revealed that fragmentation of physical activity, often measured by the active-to-sedentary transition probability, is linked to greater fatigability (Palmberg et al., 2020; Schrack et al., 2019). An earlier study found that among older people with high perceived fatigability, activity counts were lower throughout the day compared to those with low fatigability (Wanigatunga et al., 2018). Additionally, higher physical fatigability in older adults has been associated with a disrupted rest–activity rhythm, characterized by more variability, delayed transitions, and a general dampening of activity patterns (Graves et al., 2021). These findings highlight how fatigability not only diminishes overall activity levels but also disrupts the regularity and flow of movement patterns (Graves et al., 2021). However, studies targeting the diurnal patterns of strength-demanding activities related to fatigability are limited, even though such activities may be particularly sensitive to fatigue accumulation due to their reliance on neuromuscular capacity (Paris et al., 2022).

STS transitions are among the most frequent, strength-demanding movements required for daily functioning among community-dwelling older adults (Dall & Kerr, 2010; Ploutz-Snyder et al., 2002) and are challenging for balance, even posing a risk of falls (Hill et al., 2013). STS transitions play a critical role in enabling independent living, as they are necessary for completing essential daily activities, such as using the restroom or getting out of bed (Williamson & Fried, 1996). The volume and velocity of free-living STS transitions have previously been shown to associate with laboratory-based outcomes, including the total completion time of the 5×STS test and maximal isometric knee extensor strength (Löppönen et al., 2022, 2023). The ability to perform STS transitions consistently and efficiently throughout the day is crucial for maintaining independence (Hughes et al., 1996). Given their importance, assessing velocity trends in free-living STS transitions (i.e., potential slowing down of strength-demanding movements throughout the day) offers a promising opportunity to identify diurnal patterns using wearable-based measurements and potentially provides valuable insights into the physical functioning of older adults.

Building on this premise, this study aims to explore diurnal trends in free-living STS transition velocity over a 24-hr cycle and how these are associated with self-reported walking fatigability, performance fatigability, and physical functioning in community-dwelling older adults. By linking diurnal patterns of free-living STS velocity to functional capacity and fatigability, the research seeks to provide insights into the utility of free-living STS velocity patterns as wearable-based indicators of diurnal fatigability and physical functioning.

## Method

### Participants and design

We used data from the AGNES study (Active Ageing - Resilience and external support as modifiers of the disablement outcome) (*n* = 1,021), which was conducted in the Gerontology Research Center (GEREC), the University of Jyväskylä (Rantanen et al., 2018). AGNES study comprises three age cohorts (75, 80, and 85 years of age) of people living independently in the city of Jyväskylä, in Central Finland. The study protocol has been published by Rantanen and colleagues (2018) and Portegijs and colleagues (2019). The study was approved by the ethical committee of the Central Finland Health Care District and executed in accordance with the principles of the Declaration of Helsinki. All participants provided written informed consent.

### Calculation of individualized STS transition velocity trends

All AGNES study participants who participated in the laboratory testing were also asked about their interest in wearing an accelerometer in free-living, which was used to quantify the volume and the intensity of free-living STS transitions. The flow chart of the study (Portegijs et al., 2019), and the methodology for detecting free-living STS transitions and quantifying their velocity have been reported elsewhere (Löppönen et al., 2021, 2023). The records were obtained between October 2017 and December 2018. Accelerometry was conducted with a thigh-worn accelerometer (tri-axial accelerometer, which sampled continuously at 100 Hz, 13-bit analog-to-digital conversion, acceleration range ±16 G, UKK RM42, UKK Terveyspalvelut Oy, Tampere, Finland), taped on the anterior aspect of the dominant thigh using a transparent adhesive film for waterproofing (Vähä-Ypyä et al., 2015, 2018). Seven consecutive days of monitoring were conducted following a home interview (*n *= 486). Of these, 479 participants provided at least three valid monitoring days (24 hr). Across the three analyses presented, the total number of participants ranges from 423 to 478 due to differences in missing data.

**Figure 1:**
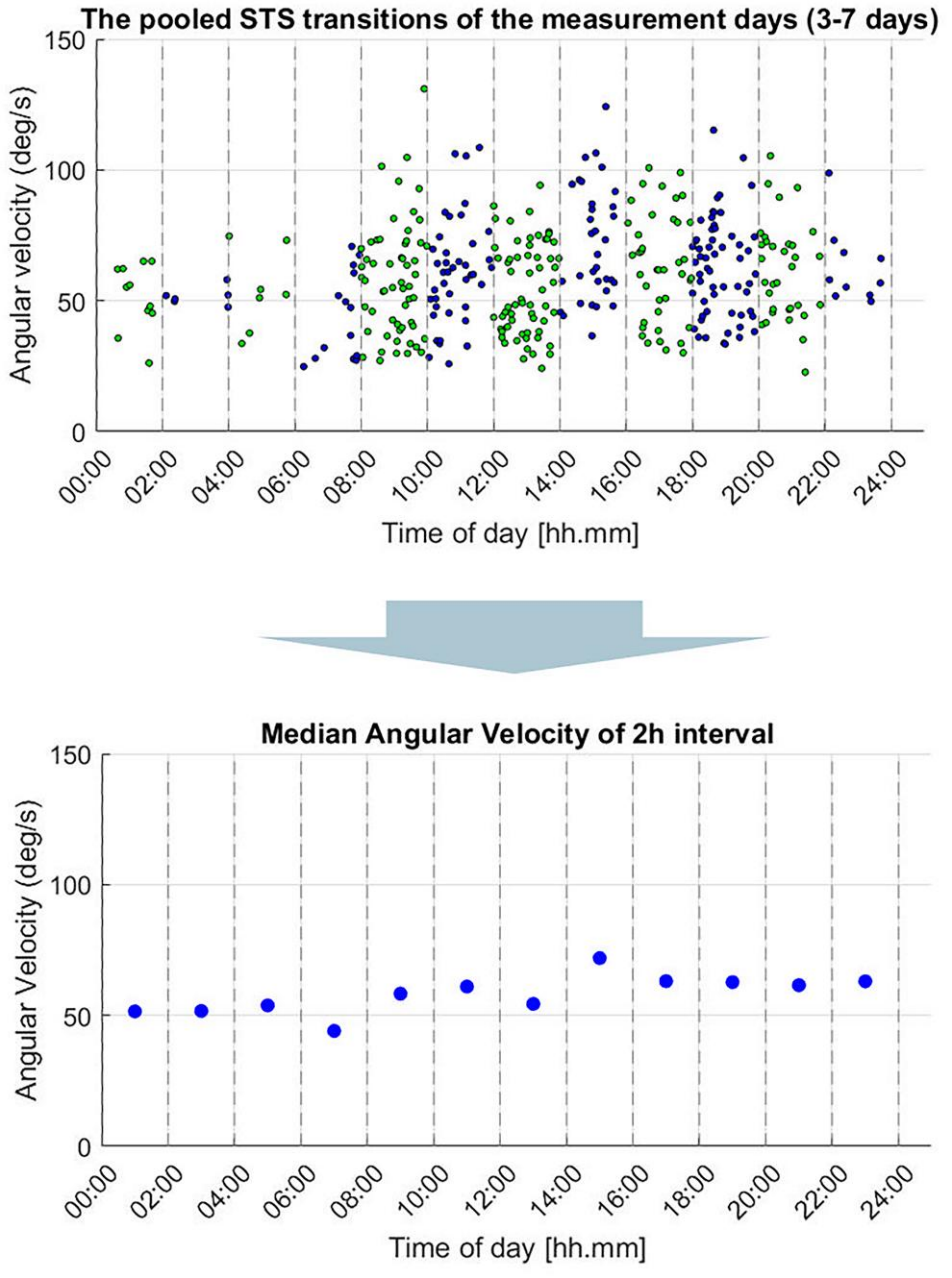
Calculation of individualized velocity trends for free-living sit-to-stand (STS) transitions.

Figure 1 shows simulated data from one typical participant’s STS transitions in the study. The STS transitions were pooled according to the time of day (from 3 to 7 measurement days) onto a single 24-hr timeline. The median values of the thigh angular velocity during the STS transitions were then calculated in 2-hr interval slots. A 2-hr interval was chosen to ensure there were enough STS transitions to calculate a median value for each interval.

As the number of STS transitions was low during nighttime, this study focused on daytime STS behavior between 08:00 and 22:00. The 24-hr trends are presented in Supplementary Figures 1–3. In the fatigability analyses, STS transitions were normalized to each individual’s daily means to enable the examination of diurnal patterns at the individual level. This approach allows for an individualized assessment of changes throughout the day, accounting for variations in average performance.

### Outcome variables

#### Self-reported walking fatigability

The self-reported fatigability during walking was assessed with the question, “How fatigued do you feel after walking for 1 hour?” Participants rated their fatigue on a 6-point scale (0 = not fatigued at all, 5 = extremely fatigued) (Yang & Wu, 2005). Based on their responses, participants were categorized into three groups: not fatigued at all (0), mildly fatigued (1–2), and highly fatigued (3–5) based on the distribution.

#### Performance fatigability

Performance fatigability severity was evaluated based on changes in walking pace during the 6-min walk test (6MWT). During the 6MWT, participants walked in an indoor corridor at the research center, walking repeated 40-m laps for 6 minutes at a self-selected walking speed. Participants were permitted to use a walking aid if necessary (Holland et al., 2014). To reduce the influence of potentially faster pacing at the beginning and slowing near the end, we compared the duration of the second-to-last 40-m lap to the second lap (Simonsick et al., 2014). Performance fatigability severity was then calculated using a modified version of the method validated for the 6MWT (Murphy et al., 2017). We first calculated the ratio of the second-to-last lap time to the second lap time to quantify within-test slowing. We then further divided the result by the total distance walked (m) during the 6MWT to place the change in performance in the context of overall walking activity. The result was further multiplied by 1,000 for reporting purposes (Palmberg et al., 2020). A higher score indicates greater slowing relative to walking speed. Participants were categorized into three equally sized tertiles (low, middle, and high).

#### Physical functioning

Physical functioning was assessed using the Short Physical Performance Battery (SPPB), which includes tests of balance, walking speed, and the 5-time sit-to-stand (5×STS) test (Guralnik et al., 1994). Each part is scored on a scale from 0 to 4, with a maximum total score of 12 points. A higher score indicates better physical functioning. Participants were categorized into three groups: high (12), middle (11–10), and low (9 and below) physical functioning based on previous studies (Bergland & Strand, 2019; Bindawas et al., 2015; Löppönen et al., 2023).

### Descriptive characteristics and other measurements

Descriptive information on participant characteristics was collected to contextualize the sample. Demographic data on age and sex were obtained from the Finnish Digital and Population Data Services Agency. Educational attainment, used as an indicator of socioeconomic status, was based on self-reported years of education (Rantanen et al., 2018). Cognitive function was measured using the Mini-Mental State Examination (MMSE), a 30-point screening tool consisting of 19 items, where higher scores reflect better cognitive performance (Folstein et al., 1975). Depressive symptoms were assessed with the 20-item Center for Epidemiologic Studies Depression scale (CES-D), with total scores ranging from 0 to 60; higher scores indicate more pronounced depressive symptoms (Radloff, 1977). Physical activity was assessed from accelerometer monitoring as the daily average mean amplitude deviation (MAD) calculated in 5-second epochs (Rowlands, 2018). Moderate-to-vigorous physical activity (MVPA) minutes were defined as the total daily minutes during which the mean amplitude deviation (MAD) (Vähä-Ypyä et al., 2015) of non-overlapping 5-s epochs exceeded 0.175 G (Karavirta et al., 2025).

### Statistical analysis

The descriptive data were reported as means and *SD*. Only participants with complete data were included in the analyses. The group‑level analysis of individual STS transition trends (2‑hr median intervals) reported the mean and confidence intervals using the summarySE function in R. Non‑parametric tests, specifically the Mann–Whitney test (wilcox.test) and Kruskal–Wallis test, were used for group comparisons, as the Smirnov–Kolmogorov test and visual inspection revealed that parametric tests were not applicable to the data. Based on previous studies reporting correlations of approximately 0.30 between free-living and laboratory-based sit-to-stand measures (Löppönen et al., 2022), a sample size of 84 participants would be required to achieve 80% power at α = 0.05. The present sample exceeds this requirement.

In the STS trend analyses, normalized free-living STS velocity between 06:00 a.m. and 10.00 p.m. was examined. Piecewise segmented regression was applied to the group-level means using the “lm” and “segmented” functions in the R-program, without specifying a predefined breakpoint. This approach allowed for the identification of potential inflection points in the data, providing insights into temporal trends in free-living STS angular velocity throughout the day. In addition, binary logistic regression models were used to evaluate the ability of normalized free-living STS velocity to differentiate between high and low classification groups (*self-reported walking fatigability, performance fatigability severity, and physical functioning*) across different time points. The odds ratio (OR) was used as a measure of effect size, reflecting the magnitude of association between STS velocity and group classification. All models were adjusted for age cohort and sex. As a sensitivity analysis, the models were additionally adjusted for the number of chronic conditions, cognitive function assessed using the MMSE, and body mass index (BMI). For analyses stratified by walking difficulties, further adjustment was made for lower-extremity functional capacity using the five-time sit-to-stand test.

Statistical significance was set at *p *< .05. The figures were created using the R statistical environment (version 4.3.3) (R Core Team, 2021) and statistical analysis with SPSS statistical software package (IBM SPSS Statistics Version 30.0.0.0; IBM Corp, Armonk, NY) (SPSS, 2021).

## Results

Descriptive statistics are presented in Table 1, stratified by self-reported walking fatigability. Age, depressive symptoms, total SPPB score, 5 × STS subtest, and physical activity differed between the groups (*p *< .001).

**Table 1:** Participant characteristics by self-reported walking fatigability (mean ± *SD*).

Variables	All (*n *= 479)	Low (*n *= 157)	Middle (*n *= 257)	High (*n *= 49)	*p*-value
**Women (%)**	60%	54.8%	61.5%	69.4%	.148
**Age (years)**	78.2 ± 3.4	77.8 ± 3.3	78.1 ± 3.2	80.1 ± 4.0	<.001
**Years of education**	11.6 ± 4.3	12.0 ± 4.7	11.5 ± 4.1	11.3 ± 4.0	.729
**MMSE (points)**	27.4 ± 2.3	27.5 ± 2.6	27.5 ± 2.1	27.1 ± 2.5	.349
**CES-D (points)**	7.8 ± 6.7	6.4 ± 6.2	8.1 ± 6.8	10.6 ± 6.5	<.001
**SPPB total score (points)**	10.4 ± 1.8	11.0 ± 1.3	10.4 ± 1.7	8.6 ± 2.5	<.001
**5×STS test time (s)**	12.4 ± 3.6	11.5 ± 3.2	12.5 ± 3.4	15.2 ± 4.8	<.001
**Mean STS velocity (deg/s)**	57.4 ± 8.7	57.7 ± 8.0	57.8 ± 9.0	54.5 ± 9.1	.014
**Mean MAD (mG)**	24.3 ± 0.8	27.3 ± 0.9	23.9 ± 0.7	16.7 ± 0.5	<.001
**MVPA (min/wk)**	221 ± 164	279 ± 119	213 ± 140	80 ± 83	<.001

*Note.* CES-D = Center for Epidemiologic Studies Depression scale; MAD = mean amplitude deviation; MMSE = Mini-Mental State Examination; MVPA = moderate-vigorous physical activity; SPPB = Short Physical Performance Battery; STS = sit-to-stand. *p*-value is based on the Kruskal–Wallis test for differences between the low, middle, and high groups.

**Figure 2:**
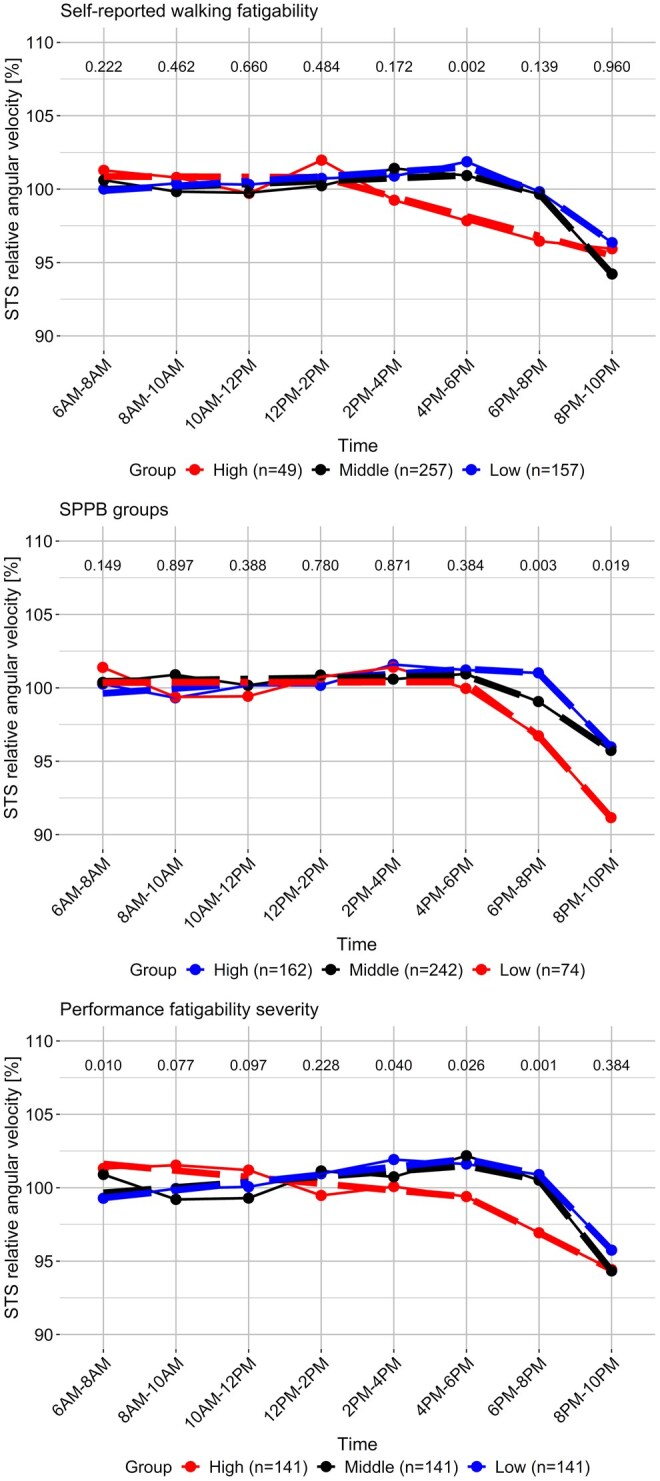
Normalized free-living sit-to-stand (STS) angular velocity across a 24-hr cycle in 2-hr intervals, compared between groups based on self-reported walking fatigability, performance fatigability severity, and physical functioning. *Note*. SPPB = Short Physical Performance Battery. A segmented regression line (without a defined breakpoint) is fitted to illustrate the downward trend of the STS angular velocity. Above this, a row displays the *p*-values from the Mann–Whitney *U*-test (Wilcoxon rank-sum test) comparing high vs low groups.

Normalized daytime trends of free-living STS angular velocity between 6:00 a.m. and 10:00 p.m. are presented in Figure 2. Individuals in the group of high self-reported walking fatigability showed significantly lower STS angular velocity between 4:00 p.m. and 6:00 p.m. (*p *< .05) compared to those in the low fatigability group. Additionally, individuals with low physical functioning demonstrated reduced STS angular velocity slightly later in the evening, between 6:00 p.m. and 8:00 p.m., and between 8:00 p.m. and 10:00 p.m. (*p *< .05), compared to those with high physical functioning. Participants in the group of highest performance fatigability (i.e., the top tertile) also exhibited lower STS angular velocity between 2:00 p.m. and 4:00 p.m., 4:00 p.m. and 6:00 p.m., 6:00 p.m. and 8:00 p.m., as well as earlier in the morning between 6:00 a.m. and 8:00 a.m. (*p *< .05), compared to those in the group of lowest performance fatigability.

**Figure 3:**
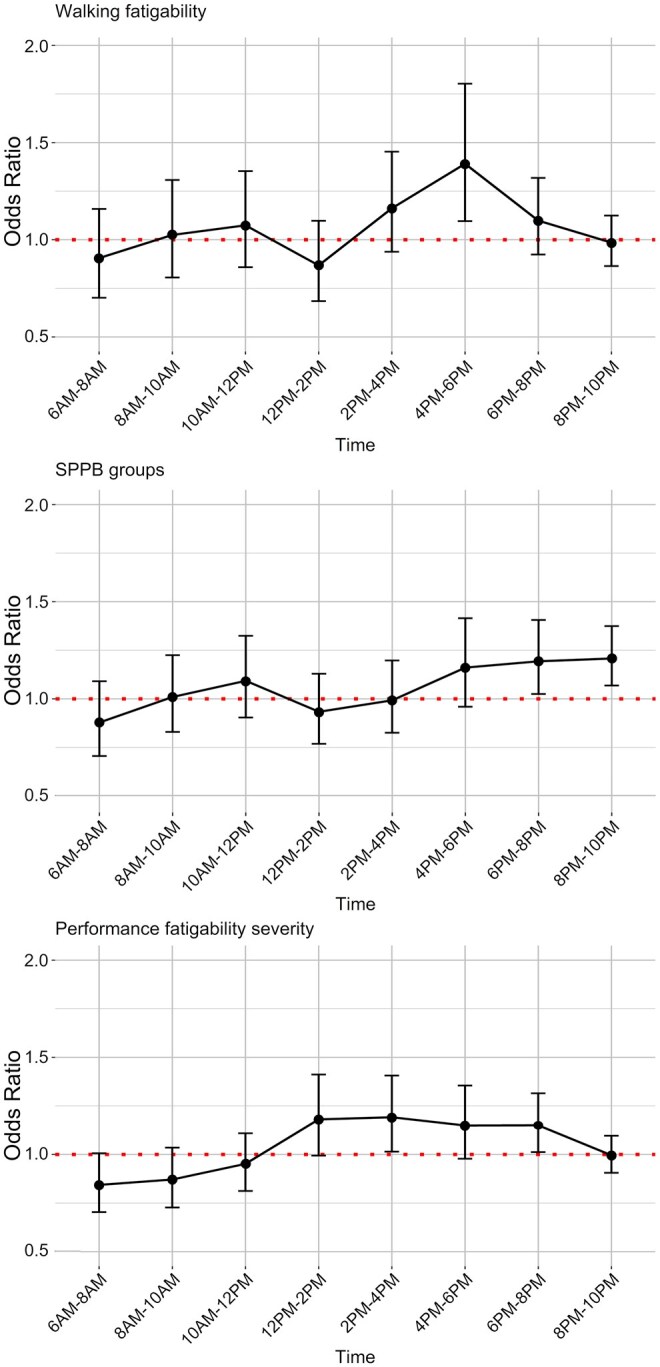
Adjusted odds ratios (95% confidence intervals) for higher normalized free-living sit-to-stand angular velocity across a 24-hr cycle in 2-hr intervals, comparing high vs low groups based on self-reported walking fatigability, performance fatigability severity, and physical functioning. *Note*. SPPB = Short Physical Performance Battery. Models adjusted for sex and age cohort.

Individuals reporting high walking fatigability exhibited significantly lower normalized free-living STS angular velocities in the late afternoon compared to those with low walking fatigability. Specifically, between 4:00 p.m. and 6:00 p.m., the OR was 1.39 per 5% decrease (*p *= .009). Similarly, individuals with high performance fatigability demonstrated lower velocities in the afternoon and early evening compared to those with low performance fatigability. Between 2:00 p.m. and 4:00 p.m., the OR was 1.19 (*p *= .034), and between 6:00 p.m. and 8:00 p.m., the OR was 1.15 (*p *= .035). Evening declines were also more pronounced among individuals with lower SPPB scores compared to those with higher scores. Between 6:00 p.m. and 8:00 p.m., the OR was 1.19 (*p *= .028), and between 8:00 p.m. and 10:00 p.m., the OR was 1.21 (*p *= .003) (Figure 3).

In the sensitivity analyses, additional adjustment for cognitive function, chronic conditions, and BMI did not materially change the risk estimates for self-reported walking fatigability and physical functioning limitations, but widened the 95% confidence intervals due to reduced statistical power, while the results remained statistically significant. However, for performance fatigability, the association attenuated in the evening and appeared to shift toward the early afternoon (12–4 p.m.). The results are presented in Supplementary Figure 6.

## Discussion

In this study, we examined diurnal trends in free-living STS transition velocity across groups defined by fatigability and physical functioning. We observed that individuals with high self-reported walking fatigability, performance fatigability, and functional limitations exhibited a declining trend in normalized free-living STS velocity as the day progressed toward the evening. Notably, individuals with higher fatigability demonstrated an earlier onset of decline in STS velocity, whereas those with poorer physical functioning exhibited a steeper decline, resulting in larger between-group differences during the evening hours. This finding suggests that monitoring free-living STS transitions could provide valuable insights into how fatigability can alter daily performance velocity of strength-demanding activities, potentially reflecting underlying physical limitations and fatigue-related challenges in daily functioning. Free-living STS transition patterns can serve as a potential novel indicator of fatigability and physical functioning in older adults.

To the best of the authors’ knowledge, no previous research has examined the association between fatigability and strength-demanding free-living activities, such as STS transitions, nor with other commonly used physical activity metrics. Previous studies have primarily focused on diurnal profiles of duration- or volume-based measures, such as device-measured step counts, activity counts, and MVPA minutes. These studies have found that activity levels peak in the morning and midday, and gradually decrease throughout the day, while sedentary time increases and reaches its highest levels in the evening (Davis & Fox, 2007; Mai et al., 2014; Sartini et al., 2015). Similar patterns were observed in the present study, with MVPA minutes peaking between 10:00 a.m. and 2:00 p.m. and decreasing toward the evening (see Supplementary Figures 4 and 5). In addition, some studies have examined how breaks in sedentary time occur throughout the day and their association with functional capacity. For example, Lai et al. (2023) found that more frequent breaks from sedentary behavior, especially in the evening, were associated with greater lower-extremity strength in older adults. (Lai et al., 2023). Nonetheless, Wanigatunga and colleagues found that older adults with high perceived fatigability had lower physical activity counts throughout the day compared to those with low fatigability, whereas those with intermediate fatigability exhibited higher activity counts early in the day compared to those with high fatigability but showed a declining trend toward the evening (Wanigatunga et al., 2018). Unlike traditional device-based metrics of physical activity such as step counts, sedentary time or MVPA minutes, which may cluster earlier in the day due to individual preferences or daylight availability (Davis & Fox, 2007; Mai et al., 2014; Sartini et al., 2015), STS transitions tend to be essential throughout the entire day, for example, due to routine activities such as toilet visits (Hughes et al., 1996). This is reflected in the distribution of the number of STS transitions across the day, as shown in Supplementary Figure 1.

The comprehensive review of Adão Martins and colleagues (2021) highlighted that the majority of objective fatigability assessment methods, particularly those targeting neuromuscular and physiological fatigue, have been developed and applied in laboratory settings (Adão Martins et al., 2021). Furthermore, the assessment of performance fatigability, such as performance decrements during standardized walking tests, also typically requires laboratory or research center environments. Therefore, there is a clear demand for the development and implementation of reliable models in free-living environments, especially considering that sensor technology is now easily accessible. However, we cannot be certain whether the observed decline in STS velocity over the course of the day is caused by fatigability. Nevertheless, the demonstrated association between lower STS velocity and greater fatigability lays the groundwork for future research exploring whether STS transitions could serve as a useful indicator for assessing performance fatigability. The device-based approach of the current study provides an excellent opportunity to study free-living fatigability indicators and can be expanded to other (clinical) populations. For example, our device-based free-living approach may be used alongside self-reported fatigability measures to investigate the severity of relapses in individuals with multiple sclerosis (Luostarinen et al., 2023). While STS transitions are commonly studied due to their concentric strength demands, the reverse movement, standing to sitting, also involves eccentric strength and neuromuscular control. Future research should explore this transition as it may provide valuable insights into fall risk and functional capacity in older adults, requiring the development and validation of appropriate assessment methods.

The observed earlier and steeper decline in free-living STS angular velocity toward the evening may reflect multiple, interacting mechanisms rather than a single cause. One possibility is fatigue accumulation over the day, which can impair neuromuscular performance, slow reaction times, and reduce coordination, thereby limiting the speed of STS transitions (Allman & Rice, 2002). Another perspective is the energetic cost-to-capacity ratio framework, which suggests that with aging, the energy demand of mobility tasks approaches or exceeds physiological capacity, increasing fatigability and potentially leading to fragmented activity patterns (Schrack et al., 2010; Schrack et al., 2020b). Applied to STS, evening declines could indicate a cumulative mismatch between energy expenditure and capacity. However, systemic energy availability alone is unlikely to fully explain these changes, as whole-body energy stores remain abundant and STS is a brief task. Instead, localized mechanisms such as diminished ATP resynthesis, reduced creatine phosphate availability, or impaired excitation–contraction coupling might contribute, alongside central factors like altered motor unit recruitment or reduced cortical drive (Chan et al., 2000; Gandevia, 2001; Lanza et al., 2005; Smith et al., 2025).

Some limitations should be considered when interpreting the findings of this study. First, the cross-sectional design does not allow for longitudinal conclusions on whether free-living STS patterns predict fatigability metrics. Second, while the sample size was reasonably large, participation in device-based measurements tends to attract a selective group, potentially reducing the generalizability of the findings. Moreover, the discrepancy in the size of the high fatigability group between self-reported and performance-based measures should be acknowledged. Finally, self-reported fatigability was assessed using a single walking-related item from a questionnaire validated in a younger population, which may limit the precision among older adults. However, these findings were consistent with those derived from the performance-based fatigability assessment, supporting the present findings despite limitations in the self-reported measure. Despite these limitations, studying has notable strengths. The extended monitoring period captures the daily variation of physical activity behaviors. Free-living STS transitions are crucial for independent living, and their frequency is less influenced by motivation or leisure preferences compared to metrics such as MVPA. Finally, the study successfully recruited a relatively large population-based sample of community-dwelling older adults, allowing for a sufficiently large sample for analysis of this demographic group.

To support clinical practice, these findings suggest several practical applications. For patient groups experiencing fatigue, such as individuals with multiple sclerosis, understanding time-of-day variations may help optimize the timing of daily activities and exercise. Furthermore, recognizing when sit-to-stand transitions slow down in older adults could be important for fall-risk assessment. Finally, these patterns may be useful for monitoring progress during rehabilitation, where changes in performance over time could indicate improvements beyond diurnal fluctuations. Finally, this study highlights the translational value of wearable technology for capturing sit-to-stand performance in free-living environments. Demonstrating the feasibility of this approach may support efforts to extend mobility assessment beyond controlled settings and inform future use of wearable sensor data to better understand free-living functional challenges among community-dwelling older adults.

## Conclusions

In this study, we observed that individuals with high fatigability and functional limitations exhibited a declining trend in normalized STS transition velocity as the day progressed toward the evening. The current study provides a unique contribution to understanding diurnal strength-demanding activity patterns among community-dwelling older adults perceiving greater fatigability. Device-based monitoring of diurnal patterns in strength-demanding activity can enhance understanding of how fatigability may alter daily activity behavior, thereby creating opportunities to promote the suitable timing of physical activity and support independent living. In addition, future studies should further examine the feasibility of monitoring trends in STS transitions as a potential indicator of fatigability.

## Supplementary material

Supplementary material is available at *Innovation in Aging* online.

## Supplementary Material

igag040_Supplementary_Data

## Data Availability

After completion of the study, data will be stored at the Finnish Social Science Data Archive without potential identifiers (open access). Until then, pseudonymized datasets are available to external collaborators subject to agreement on the terms of data use and publication of results. To request the data, please contact Professor Taina Rantanen (taina.rantanen@jyu.fi).
