# Supplementary material for: Does sit-to-stand transition velocity vary across the day? Association with physical functioning and fatigability in community-dwelling older adults
Source: Innov Aging. 2026 Apr 16;10(6):igag040. doi: 10.1093/geroni/igag040 (PMC13186197; doi:10.1093/geroni/igag040)
Supplement: igag040_Supplementary_Data [file igag040_supplementary_data.zip › Innage_suppl_Löppönen_et al.pdf]

Supplementary Material: Löppönen et al. Does sit-to-stand transition velocity vary across the day? Association with physical functioning and fatigability in community-dwelling older adults.

Supplementary Figure 1. Number of STS transitions in two-hour intervals in relation to self-reported fatigability, performance fatigability, and physical functioning limitations based on SPPB scores.

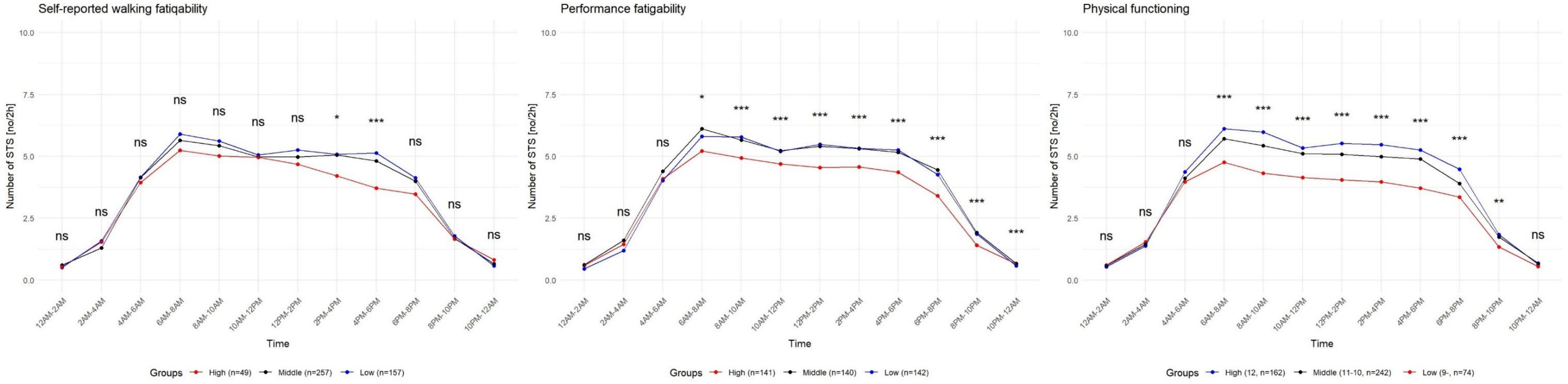

Note. Group comparisons (all three groups) were conducted using the independent-samples Kruskal–Wallis test. Asterisks denote significance levels: *p < 0.05; **p < 0.01; ***p < 0.001.

Supplementary Figure 2. Absolute angular velocity of STS transitions in two-hour intervals in relation to self-reported fatigability and physical functioning limitations based on SPPB scores.

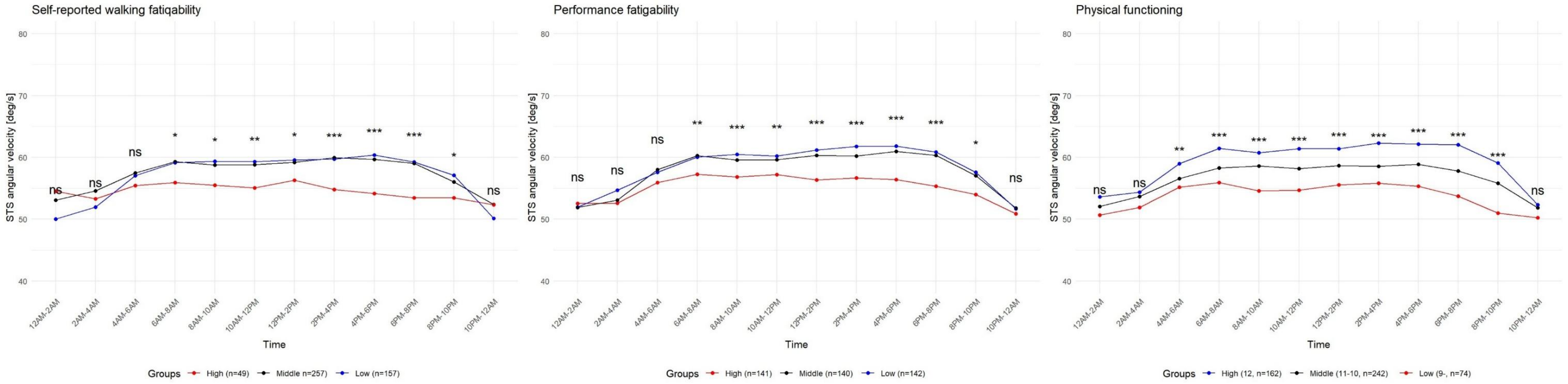

Note. Group comparisons (all three groups) were conducted using the independent-samples Kruskal–Wallis test. Asterisks denote significance levels: *p < 0.05; **p < 0.01; ***p < 0.001.

Supplementary Figure 3. Angular velocity of STS transitions relative to daily averages in two-hour intervals in relation to self-reported fatigability and physical functioning limitations based on SPPB scores.

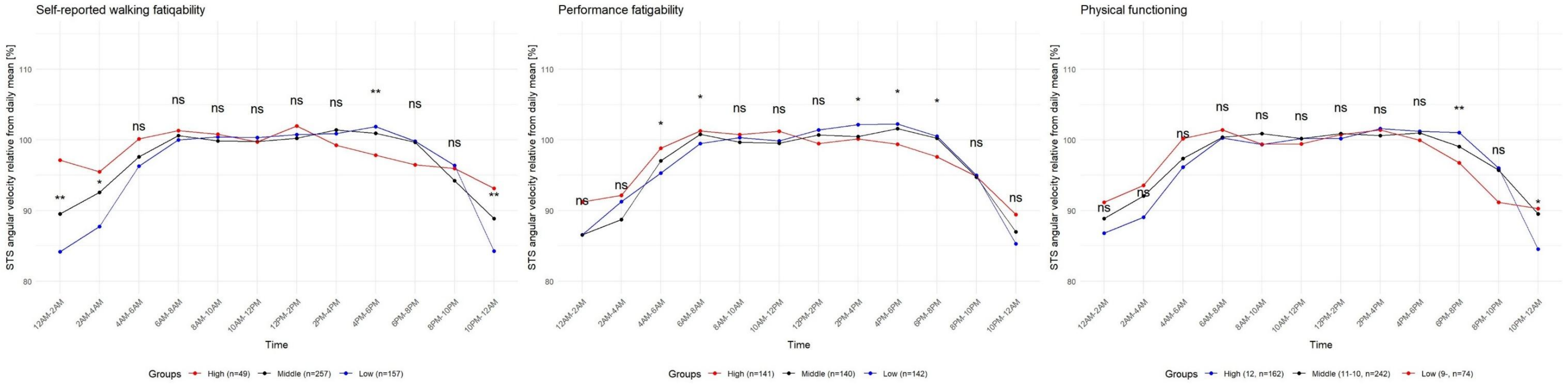

Note. Group comparisons (all three groups) were conducted using the independent-samples Kruskal–Wallis test. Asterisks denote significance levels: *p < 0.05; **p < 0.01; ***p < 0.001.

Supplementary Figure 4. Moderate-vigorous physical activity minutes in two-hour intervals in relation to self-reported fatigability and physical functioning limitations based on SPPB scores.

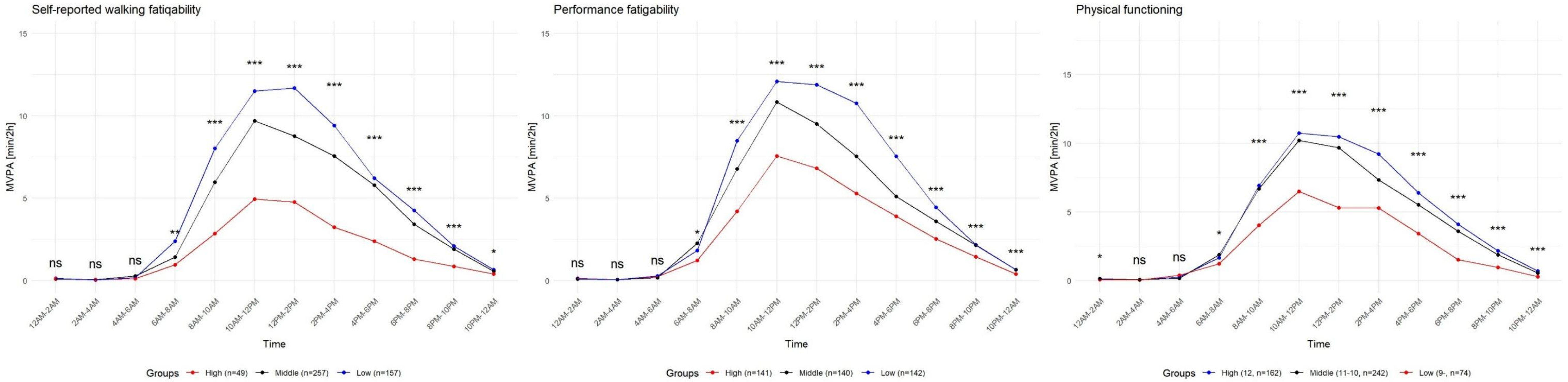

Note. The MVPA threshold was defined as 0.175 g mean amplitude deviation (MAD) calculated over 5-second epochs, following Karavirta et al. (2025). Group comparisons (all three groups) were conducted using the independent-samples Kruskal–Wallis test. Asterisks denote significance levels: *p < 0.05; **p < 0.01; ***p < 0.001.

Karavirta, L., Aittokoski, T., Pynnönen, K., Rantalainen, T., Westgate, K., Gonzales, T., Palmberg, L., Neuvonen, J., Lipponen, J. A., Turunen, K., Nikander, R., Portegijs, E., Rantanen, T., & Brage, S. (2025). Physical determinants of daily physical activity in older men and women. *PLoS One*, *20*(2), e0314456. https://doi.org/10.1371/journal.pone.0314456

Supplementary Figure 5. Absolute MAD of the highest one-minute period (i.e., sum of twelve 5-second bouts; unit: g) for each two-hour interval across groups defined by self-reported fatigability and physical functioning limitations based on SPPB scores.

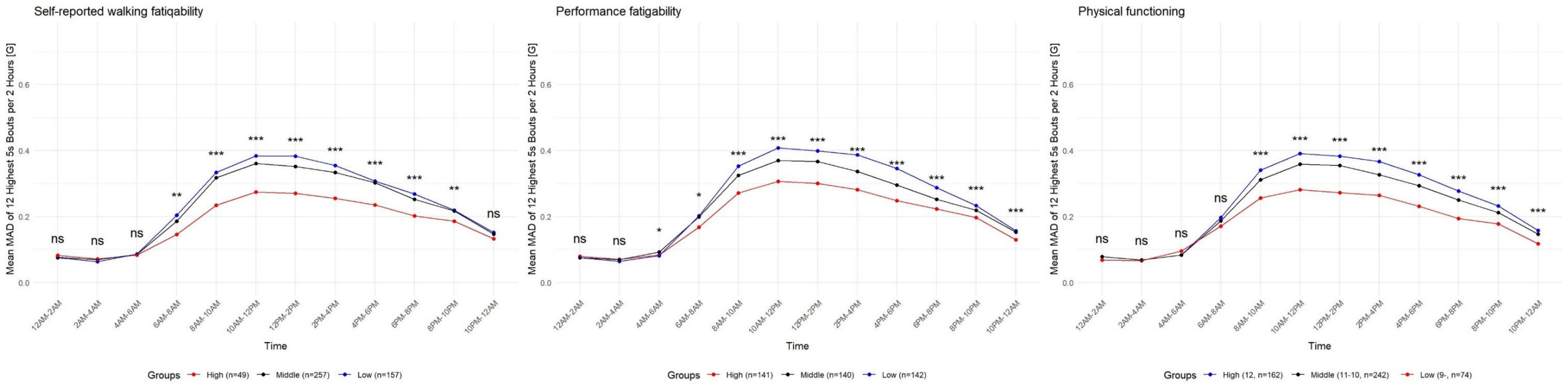

Note. Group comparisons (all three groups) were conducted using the independent-samples Kruskal–Wallis test. Asterisks denote significance levels: *$p < 0.05$; **$p < 0.01$; ***$p < 0.001$.

Supplementary Figure 6. Sensitivity analyses of logistic regression models.

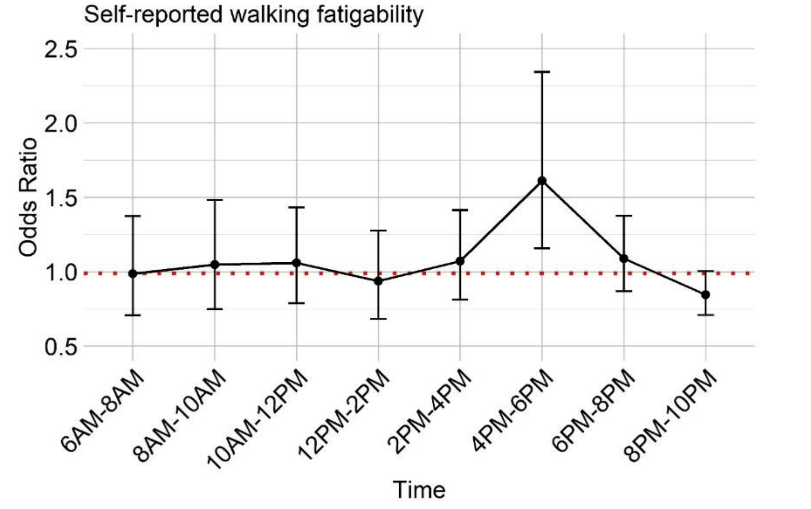

Adjusted for age, sex, five-time sit-to-stand test time, number of diseases, body mass index, and Mini-Mental State Examination.

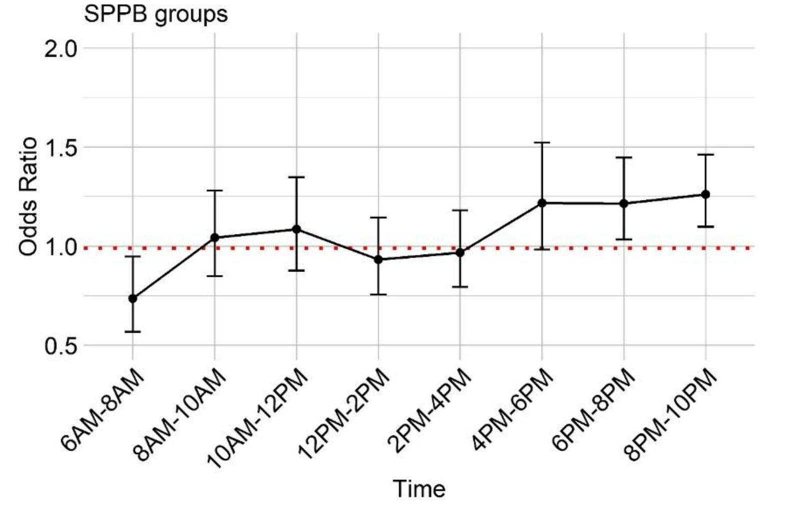

Adjusted for age, sex, number of diseases, body mass index, and Mini-Mental State Examination.

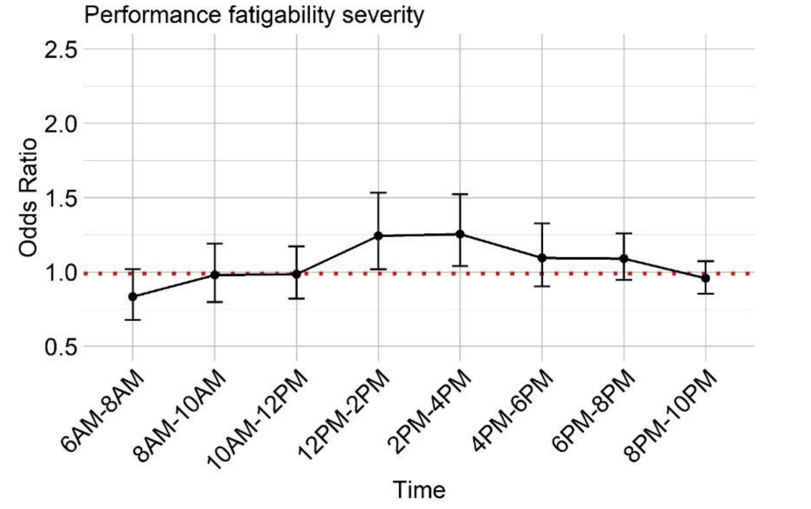

Adjusted for age, sex, number of diseases, body mass index, and Mini-Mental State Examination.
